# Dissecting and Recording from The C. Elegans Neuromuscular Junction

**DOI:** 10.3791/1165

**Published:** 2009-02-25

**Authors:** Janet Richmond

**Affiliations:** Department of Biological Sciences,

## Abstract

Neurotransmission is the process by which neurons transfer information via chemical signals to their post-synaptic targets, on a rapid time scale.   This complex process requires the coordinated activity of many pre- and post-synaptic proteins to ensure appropriate synaptic connectivity, conduction of electrical signals, targeting and priming of secretory vesicles, calcium sensing, vesicle fusion, localization and function of postsynaptic receptors and finally, recycling mechanisms.  As neuroscientists it is our goal to elucidate which proteins function in each of these steps and understand their mechanisms of action.  Electrophysiological recordings from synapses provide a quantifiable read out of the underlying electrical events that occur during synaptic transmission.  By combining this technique with the powerful array of molecular and genetic tools available to manipulate synaptic proteins in *C. elegans*, we can analyze the resulting functional changes in synaptic transmission.

The *C. elegans* NMJs formed between motor neurons and body wall muscles  control locomotion, therefore, mutants with uncoordinated locomotory phenotypes (known as *unc* s) often perturb synaptic transmission at these synapses ^1^.  Since *unc* mutants are maintained on a rich supply of a bacterial food source, they remain viable as long as they retain some pharyngeal pumping ability to ingest food.  This, together with the fact that *C. elegans* exist as hermaphrodites, allows them to pass on mutant progeny without the need for elaborate mating behaviors.  These attributes, coupled with our recent ability to record from the worms NMJs ^2,3,7^ make this an excellent model organism in which to address precisely how *unc* mutants impact neurotransmission.

The dissection method involves immobilizing adult worms using a cyanoacrylic glue in order to make an incision in the worm cuticle exposing the NMJs.  Since *C. elegans* adults are only 1 mm in length the dissection is performed with the use of a dissecting microscope and requires excellent hand-eye coordination.  NMJ recordings are made by whole-cell voltage clamping individual body wall muscle cells and neurotransmitter release can be evoked using a variety of stimulation protocols including electrical stimulation, light-activated channel-rhodopsin-mediated depolarization ^4^ and hyperosmotic saline, all of which will be briefly described.

**Figure:**
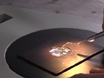


## Protocol

### A. Preparation of tools used for the dissection.

1. The dissection/recording chamber: We typically construct our recording chamber out of a 1/16^th^ inch magnetic sheet, with a circular hole drilled into the center that is large enough to accommodate a 22 mm diameter circular cover glass.  The outer dimensions of the chamber will depend on the microscope stage used to do the recordings.  On the reverse side of the magnetic sheet , we attach a 48 x 60 mm cover slip, which we adhere to the chamber by placing a pellet of low melt Paraplax tissue embedding wax in each corner of the cover slip sandwiched between the glass and the chamber.  The wax is then melted on a hot plate,  glass plate facing up, until it forms a continuous layer gluing the cover slip to the chamber.  Do not leave this step unattended as the wax melts quickly.  The chamber should be removed and cooled as soon as the wax has melted.  Excess wax that has seeped along the inner edge of the 22mm circular cavity can be removed using a razor blade edge.
2. Sylgard-coated cover slips: The dissection is performed on a 22 mm, Sylgard-coated cover slip that is placed within the circular cavity of the recording chamber.  The Sylgard coat helps the cyanoacrylic glue adhere to the cover slip when gluing the worms down and also acts as a cushion on which to break back the glue pipette tip when it becomes plugged.  Sylgard is made up fresh according to  the manufactures instructions (10 parts silicone base to 1 part curing agent by weight).  A small drop of Sylgard is then placed on each cover slip and smeared across using a razor blade edge to make an even surface.  The coated cover slips are placed in large flat covered containers and left in a 65° degree oven overnight to cure.
3. Pipettes: Typically, we use the same 1 mm outer diameter filament borosilicate glass to generate the pipettes for gluing worms down, making cuticle incisions and recording from muscles, using an electrode puller.  It is advisable to pull a jar of pipettes before attempting a dissection as this stage can require several rapid exchanges of pipettes.
4. Glue applicator: Glue is loaded and applied from the tip of a glue pipette, by mouth-pressure. The gluing pipette is inserted into one end of a 2 foot piece of polyethylene tubing with an inner diameter that matches the outer diameter of the pipette glass (usually 1 mm). The other end of the tubing holds an eppendorf pipette tip that acts as a mouth piece.
5. Extraction pipette: The worm viscera must be removed from the worm cavity once the cuticle incision has been made.  This is achieved with a piece of tubing attached to an eppendorf pipette tip similar to the glue applicator but in this case the glass extraction pipette tip is broken back sufficiently to allow eggs and viscera to be easily vacuumed out of the worm cavity.

### B. The dissection

1. In the recording chamber a circular wax pen line is drawn close to the perimeter of the chamber to which a Sylgard-coated cover slip is pressed firmly in place (Sylgard-coat facing upwards).  This wax line immobilizes the cover slip and prevents the recording solution from seeping under and dislodging the cover slip during dissection and recording.  The chamber is then filled with extracellular recording solution and several worms are placed in the center using a worm pick.  Worms will swim in solution, however with practice, these worms can be glued down without any further procedures such as cooling to prevent movement.  During the training stage, it can help to practice gluing mutant worms in which swimming is impaired (such as *unc-31* mutants).
2. A glue container fashioned from a PCR cap held in wax or modeling clay, is used to hold a working stock of the cyanoacrylic glue.  Glue pipettes with similar tip dimensions to the recording pipettes (normally ~4 megohms resistance) are used to apply glue.  Using the glue applicator, a small amount of glue is sucked up into the glue pipette, using the dissection scope to visualize the pipette tip as the glue is sucked up.
3. The cyanoacrylic glue (HistoacrylBlue, Aesulap) polymerizes on contact with extracellular recording solution, therefore it is important to maintain positive pressure on the glue pipette as it enters the chamber solution to prevent the glue from hardening and plugging the pipette.  As soon as the glue pipette is in the chamber solution, a small but constant stream of glue needs to be applied onto the Sylgard surface under mouth-pressure control to prevent plugging.  If the pipette becomes plugged it can sometimes be unblocked by gently tapping the tip on the sylgard-coated glass.  It is recommended that you practice gluing before attempting to glue worms.  If you can routinely write your name in glue on the Sylgard, with letters made of lines thinner than the worms width you are ready to proceed to gluing the worm cuticle.
4. To glue a worm, use positive-pressure to attach a small amount of glue to either the head or tail of the worm and rapidly draw the worm down onto the Sylgard surface.  Try to attach the worm near the center of the chamber as worms glued too near to the edge will be difficult to reach with recording pipettes.  Then apply a constant stream of glue along the dorsal edge of the worm’s cuticle from the starting attachment, forcing the worm into a C-position with the vulva facing inward (for ventral muscle recordings).  Any gaps in this glue line should be filled in so that the worm is strongly attached, to prevent the worm from detaching during the cuticle incision step.
5. To make the cuticle incision, switch to a hand-held dissection pipette.  This pipette needs to be sharper than the glue/recording pipettes and have a short, sturdy shaft.  Using the highest-magnification on the dissecting scope, align the pipette parallel to the longitudinal axis of the worm and insert the tip about midpoint along the worm (close to the vulva) making the incision at the cuticle/glue interface.  This incision releases the worms hydrostatic pressure forcing eggs and intestines out through the incision point.  Continue cutting the cuticle towards the head of the worm with a slicing motion similar to opening a letter, until you reach the pharynx.  Switch over to the extraction pipette to vacuum out the internal organs of the worm.  After clearing the incision, the opened cuticle will retain it’s cylindrical shape.  Use a fresh gluing pipette to spot weld the cut edge of the cuticle down onto the Sylgard surface.  It is important to use minimal glue spots at this stage to avoid damaging or obscuring the NMJs. The ventral nerve cord and body wall muscles are now exposed.
6. To remove the basement membrane that covers the NMJs, suck off the extracellular recording solution from the chamber and apply collagenase (0.4mg/ml in extracellular recording solution) for about 10-20 seconds; remove and wash several times with fresh extracellular recording solution.  Proceed to the recording stage.

### C. Patch-clamp recording

1. Position the recording chamber on an up-right microscope stage using a 10X objective to center the worm.  Switch to a 40X water-emersion objective with DIC and check that the body wall muscles and nerve cord are intact.  Position the worm so that the longitudinal axis of the worm is parallel with the front edge of the scope, allowing electrodes to be introduced from both the left and right sides.
2. Patch the muscle using standard patch-clamping techniques.  Recording electrodes of about 4 megohms resistance work well.  Once the electrode touches the muscle membrane, increase negative-pressure until a gigohm seal is obtained.  For the whole-cell voltage-clamp recording mode, apply more suction and zap the membrane until the gigohm seal is ruptured. In a good recording, body wall muscles of wild-type adult worms typically have a cell-membrane capacitance of ~70 pF and can be stably recorded for at least 10 minutes at a holding potential of -60 mV with minimal holding currents (~50 pA).
3. The extracellular recording solution consists of (in mM): NaCl 150, KCl 5, CaCl_2_ 5, MgCl_2_ 4, glucose 10, sucrose 5, HEPES 15 (pH 7.3, ~ 330mOsm).  The patch pipette contains (in mM): KCl 120, KOH 20, MgCl_2_ 4, (N-tris[Hydroxymethyl] mthyl-2-aminoethane-sulfonic acid) TES 5, Dihydrate CaCl_2_ 0.25, Na_2_ATP 4, sucrose 36, EGTA 5 (pH 7.2, ~312 mOsm).  Note: These solutions artificially load muscle cells with chloride, so that the GABA receptor-mediated currents are inward due to chloride efflux at a holding potential of -60 mV.  Variations on these solutions have been used (see publications for details).
4. Evoked release.  Typically we place a second electrode containing extracellular solution on the ventral nerve cord anterior to the patched muscle.  This loose patch configuration requires large depolarizing stimuli (on the order of 10s of volts) of short duration (1-2ms) to achieve maximal evoked responses (typically 2.0-2.5 nA) in wild-type worms.  The limitations of this approach are: 1) Only a few evoked responses can be delivered before the nerve cord is irreversibly damaged and 2) For unknown reasons, only cholinergic evoked responses can be elicited using this stimulation configuration.
5. Light activated channel-rhodopsin stimulation.  Recently, the Gottschalk lab has generated transgenic worms expressing algae-derived channel rhodopsins that depolarize the membrane upon light-stimulation in subsets of neurons, depending on the promoter used to drive expression ^4^.  In the case of the *C. elegans *NMJ, integrated lines expressing channel rhodopsin in either the cholinergic motor neurons or GABAergic motor neurons are now available.  Since channel rhodopsin only responds to light when all-trans retinal is associated with the opsin moiety, worms used for this application must be grown on bacteria laced with retinal.  A green light pulse can then be used to repeatedly activate the channel rhodopsin causing evoked release of either ACh or GABA, depending on which transgenic worms are used.  These transgenic worms can be crossed into mutant strains.  The advantage of this technique over the previous electrical stimulation include; 1) Light-activation does not cause neuronal damage, allowing many repeat stimulations to be performed and 2) Evoked release from either GABAergic or cholinergic  motor neurons can be independently studied.
6. Hyperosmotic-induced release.  To measure the size of the readily-releasable vesicle pool (reflecting the summed pool of primed vesicles from all NMJ’s onto the recorded muscle), a pressure-pipette containing hyperosmotic extracellular solution (adjusted to ~850 mOsm  by adding additional sucrose) is used.  Pressure-pulse applications of 1-3 seconds produce a barrage of asynchronous post-synaptic events using the standard solutions described above.

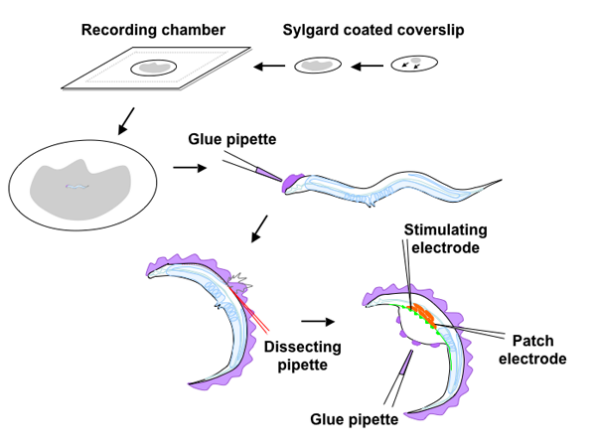


**Figure 1: Schematic overview of the *C. elegans *dissection and neuromuscular recording configuration**

A sylgard coated coverslip made by smearing a drop of Sylgard across the glass and curing overnight is placed in the circular cavity of the prefabricated recording chamber and covered with recording solution.  A worm placed in the center is glued to the Sylgard surface starting from the head or tail, applying glue to the dorsal side of the worm, using a glue pipette,.  Once gluing is complete, an incision adjacent to the vulva, at the interface between the cuticle and glue is made with a glass needle and extended toward the head (see red perforated line).  Following extraction of viscera and eggs from the body cavity,  the cut edge of the incision is spot-welded with glue to the Sylgard exposing the ventral nerve (green) and body wall muscles (red).  The ventral-medial muscle anterior to the vulva is patch-clamped and the stimulating electrode is placed upstream on the nerve cord.

### Representative results


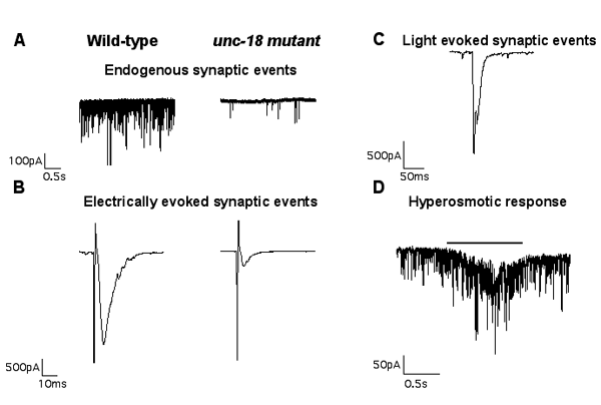


Whole-cell, voltage-clamped body wall muscle recordings are shown for each of the three stimulation protocols described above (electrically evoked, light-evoked and hyperosmotic responses).  In all cases the ventral-medial body wall muscle is clamped at -60 mV.  A. Wild-type muscle cells typically exhibit high rates of endogenous inward miniature synaptic currents in our standard recording solutions. B. A 2ms depolarization of the anterior ventral nerve cord causes synchronous release from several synapses resulting in a large evoked response of around 2-2.5 nA in wild-type worms.  Representative traces to the right of the wild-type traces in A and B show synaptic release in a severely uncoordinated mutant (unc-18).  Panel C shows a light-evoked response from the neuromuscular junction of a wild-type worm expressing channel rhodopsin in cholinergic neurons.  A 2ms light pulse typically produces a response of 1.5-1.8 nA.  D. Pressure-ejection of 850 mOsm hyperosmotic saline onto the muscle for 1s, produces an asynchronous barrage of miniature synaptic currents representing the readily-releasable pool of vesicles.

## Discussion

The genetic model organism *C, elegans * is ideally suited for the study of synaptic transmission, through the functional analysis of mutations in genes encoding synaptic proteins.  Here we have described a dissection technique that renders the *C. elegans *NMJs accessible for electrophysiological analysis.  The accompanying video depicts the critical steps in the *C. elegans *dissection and the typical recording configuration used to measure synaptic activity.  The three steps that pose  the greatest challenges in learning this technique are; 1) the worm gluing step, 2) performing a successful incision, and 3) patching on to the body wall muscles.  Once these have been mastered, this preparation is amenable to the recording of the post-synaptic muscle in either the voltage-clamp or current-clamp modes.  Responses to evoked stimuli can be readily measured using this dissected preparation in a variety of mutant backgrounds.  Given the already minute size of the wild-type adult worm (1 mm in length), synaptic mutants that do not reach adulthood or remain scrawny may not be amenable to this dissection approach.  The motor neurons of the ventral nerve cord are also very small (cell soma’s 2-5 μm) and pose additional challenges for electrophysiologists, although several labs have successfully recorded from *C. elegans *neurons in variations of the dissected preparation described here ^ 5 6^.
